# Hierarchical Silica–Cellulose Nanoarchitectures from Rice Straw: A Waste-to-Value Platform for Autonomous Osteogenic Bone Regeneration

**DOI:** 10.3390/jfb17080367

**Published:** 2026-07-30

**Authors:** Zahra Khaleghi Moghadam, Mohammad Nourany, Saadi Hosseini, Naser Farokhi, Atefeh Alipour, Pär K. Ingvarsson, Hosein Shahsavarani

**Affiliations:** 1 Department of Cell and Molecular Biology, Faculty of Life Science and Biotechnology, Shahid Beheshti University, Tehran 19839-69411, Iran; 2 Laboratory of Regenerative Medicine and Biomedical Innovations, Pasteur Institute of Iran, National Cell Bank, Tehran 13169-43551, Iran; 3 Department of Polymer Engineering and Color Technology, Amirkabir University of Technology, Tehran 15916-34313, Iran; 4 Department of Nanobiotechnology, Pasteur Institute of Iran, Tehran 13169-43551, Iran; 5 Department of Plant Biology, Swedish University of Agricultural Sciences, SE-750 07 Uppsala, Sweden

**Keywords:** rice straw, decellularised plant scaffold, silica-cellulose, autonomous osteoinduction, growth factor-free osteogenesis, hMSC differentiation

## Abstract

Current bone regeneration strategies face significant constraints, relying either on synthetic scaffolds with slow degradation that require biochemical supplements or on bioactive fillers. Recently, the focus has shifted towards functional natural biomaterials with inherent osteoinductive potential. This study presents a promising candidate based on a silica-containing plant-derived scaffold fabricated from rice straw, a sustainable resource with >20 wt.% silica. Morphological analyses revealed that the decellularised rice straw scaffold exhibits a unique surface pattern with a three-dimensional nanoarchitecture featuring parallel fibrillar protrusions and cellulosic spikes evenly distributed across the surface. Elemental mapping revealed abundant silicon, with minor traces of calcium and phosphorus, all of which are crucial components of bioactive minerals. The scaffold was highly biodegradable, with 81.7% weight loss after 90 days, attributed to its high water absorption (337%). The scaffold demonstrated excellent biocompatibility, maintaining MG63 cell viability and promoting robust adhesion and proliferation, with relative metabolic activity increasing from 105% at day 5 to 125% at day 7 relative to TCPS controls. Most remarkably, when seeded with adipose-derived human mesenchymal stem cells (hMSCs) in the absence of osteogenic medium, the scaffold induced significant biomineralisation. This osteoinductive capacity is attributed to its unique surface pattern, high roughness, and polar cellulosic substrate, together with bioactive silica that releases soluble silicon species. This work represents how agricultural waste can be upcycled for autonomous bone regeneration.

## 1. Introduction

Bone injuries and damage alongside skeletal disorders are a major part of global health issues, with more than two million bone grafting procedures being performed annually worldwide to treat critical-sized osseous defects [1]. These injuries can arise from accidents, infection, tumours, and congenital disorders, which often require surgical intervention to stimulate bone repair and regeneration [2]. Additionally, bone-related chronic diseases like osteoporosis and osteoarthritis afflict nearly 200 million people globally [3]. With the global rise in the elderly population and the incidence of bone-related diseases and damage, the demand for effective treatments is projected to rise substantially in the coming decades [4,5]. The intrinsic regenerative capacity of bone is limited, and the processes involved in natural healing are often inadequate to repair critical defects beyond a certain size threshold [6]. To address these challenges, bone grafting has become a standard surgical technique to facilitate skeletal reconstruction [7,8,9]. The existing graft options have significant drawbacks that limit their effectiveness and application. Autografts, for example, as the gold standard, utilise a patient’s own bone and require invasive harvesting and are constrained by limited bone supply [10]. Allografts and Xenografts also pose the risk of disease transmission and contamination [11].

Due to the limitations of these conventional procedures, the development of bioengineered graft substitutes has become a major research priority in bone tissue engineering and regenerative medicine. Over the past decade, researchers have focused on developing biomimetic scaffolds that recapitulate key features of the native tissue microenvironment, using renewable and sustainable resources to achieve efficient biocompatibility, biodegradability, and bioactivity [12,13]. Calcium phosphate-based biomaterials, especially those with porous architecture [14], and synthetic polymers such as polylactic acid (PLA) and poly (ε-caprolactone) (PCL) as carriers of osteoinductive compounds [15] have been commonly employed as active ECMs. These biomaterials possess unique properties but also have certain disadvantages, such as solubility and biodegradability rates, acidic side products of the polymeric biomaterials, and processing requirements.

However, in recent years, cellulosic biomaterials have gained interest as biocompatible natural polymers, especially plant tissue-derived cellulosic ECMs [16]. One of the main parameters required for biomaterials to direct the fate of stem cells towards a predefined tissue is the physical cue, defined as surface roughness and organised patterns [17]. The ordered organisation of plant cells in the plant tissue has led to uniquely organised patterns once the cell lumen is removed through different decellularisation methods. The surface patterns enable the substrate to remodel cell spreading, and the nanometric surface roughness facilitates cell adhesion to the substrate and their growth [18]. More importantly, the surface physicochemistry of cellulosic biomaterials, particularly their abundant hydroxyl groups and hydrophilic nature, plays a critical role in mediating protein adsorption, which subsequently facilitates cell adhesion and spreading [19]. Various procedures and methods were developed to decellularise plant tissues as the focus on cellulosic biomaterials continued to grow. These include enzymatic, chemical, and physical methods, with chemical processes being the favourite due to their simplicity, efficiency, and low cost [20]. Regarding the natural polymers employed as scaffolds, most of them generally lack the essential bioactive components and the microenvironmental cues needed to effectively direct osteogenic differentiation, with the need for loading active compounds [16]. However, our recent investigations have shown the inherent potential of plant tissue-derived scaffolds to direct the fate of adipose-derived human mesenchymal stem cells (hMSCs) toward bone tissue, with continuous accumulation of biominerals and calcification [16,21].

Concerning the bioactive minerals commonly used in bone regeneration, silicon (Si) has emerged as an important component of inorganic bioactive compounds, capable of inducing biomineralisation and accelerating bone formation [22].

According to the literature, silica-based bioactive glasses with soluble silica enhance osteoblast activity and bone matrix deposition through the controlled release of soluble ionic products like orthosilicic acid that stimulate intracellular signalling pathways [23,24]. In addition to soluble forms, structural silica is also capable of inducing collagen synthesis and biomineralisation as the predominant inorganic constituent of bone. Thus, the presence of silica as part of the scaffold materials is considered a highly promising approach to improve bone regeneration capacity [25,26,27]. Among plant tissues, rice husks are an abundant agricultural byproduct/waste that contain 10 to 20 wt.% silica, mainly in the form of phytoliths that could provide a sustainable source of biogenic silica, with about 80% lignocellulose [28,29]. The silica exists as particles of amorphous hydrated silica gel embedded within the siliceous cell walls [30].

The use of rice straw-derived materials for bone tissue engineering has been explored primarily through extraction of nanocellulose or synthesis of ceramic particles, as summarised in Table 1. However, the potential of rice straw as a possible biomaterial candidate in its decellularised form has not yet been investigated. Therefore, in this study, the rice straw was chemically decellularised, and its potential as a bioactive scaffold containing inherent silica was investigated for bone tissue engineering applications. As part of the physical cue, the morphology and surface properties of the scaffold were characterised. The chemical structure of the decellularised plant tissue was analysed, and its composition was mapped, alongside an evaluation of the substrate’s hydrophilicity. The MG63 cell line, a human osteosarcoma cell line, was used to investigate the potential of decellularised rice straw as a biomaterial and its ability to support osteoblast viability, adhesion, and spreading. In the final step, hMSCs were seeded onto the scaffold to evaluate its potential to induce biomineralisation and direct the stem cells towards bone tissue in the absence of osteogenic media. A positive control (TCPS) containing the osteogenic differentiation factors was used for comparison.

## 2. Materials and Methods

### 2.1. Materials

Rice straw was obtained from Guilan province in northern Iran. Dulbecco’s Modified Eagle Medium (DMEM), foetal bovine serum (FBS), and antibiotics (penicillin/streptomycin) were purchased from Gibco (Waltham, MA, USA). Adipose-derived human mesenchymal stem cells (hMSCs) were provided by IBRC Cell Bank (Tehran, Iran). All plasticware for cell culture was purchased from SPL Lifesciences (Pocheon-si, Republic of Korea). TRIzol reagent, chloroform, isopropanol, and ethanol were purchased from Invitrogen-Thermo Fisher Scientific (Waltham, MA, USA) for RNA extraction. Alizarin red stain, cetylpyridinium chloride (CPC), p-nitrophenyl phosphate (pNPP), and other chemicals were purchased from Sigma-Aldrich (St. Louis, MO, USA).

### 2.2. Decellularisation and Scaffold Preparation

The decellularisation protocol used in this study was adapted from our previously established method [3], with minor modifications to suit rice straw. Rice straw pieces with dimensions of 2 cm × 0.5 cm were initially soaked in deionised water for 6 h and were later washed with it three times. In the next step, to remove the plant cells and obtain an acellular scaffold, rice straw was decellularised using the following protocol: it was first incubated in a 10% sodium dodecyl sulfate (SDS) solution at 25 °C for 5 days while on a shaker. Then, they were washed three times with deionised water, followed by incubation in 10% sodium hypochlorite containing 0.1% Triton X-100 for 2 days. In the next step, the scaffolds were washed extensively with deionised water, immersed in hexane for 1 min, and washed three more times with PBS. The decellularised scaffolds were then lyophilised and stored at room temperature until use. This decellularisation protocol effectively removed cellular material while preserving the native cellulose architecture, as previously demonstrated [3,6,13]. To evaluate the efficiency of the decellularisation process, DNA content analysis was performed before and after the process using a fluorometric assay. In this regard, 10 mg of the native and the decellularised scaffold powder was lysed, and the plant DNAs were extracted using cetyltrimethylammonium bromide (CTAB) buffer and were isolated according to the manufacturer’s protocol. The DNA concentration was measured for each sample by reading the absorbance at 260 nm using a spectrophotometer. The residual DNA content in the decellularised scaffold was reduced to well below the universally accepted threshold for successful decellularisation (<50 ng/mg dry tissue weight) [36]. Prior to UV sterilisation, scaffolds were immersed in 70% ethanol for 10 min to enhance surface wettability and eliminate contaminants, followed by washing with sterile PBS [3].

### 2.3. Scaffold Characterisation

The surface morphology of pristine rice straw and its decellularised form was evaluated using field-emission scanning electron microscopy (FESEM; TESCAN GROUP, a.s., Brno, Czech Republic). The elemental analysis of the surface was also evaluated using a Phenom Pharos desktop field-emission scanning electron microscope (SEM) equipped with an energy-dispersive X-ray spectroscopy (EDX) system (Phenom World, Eindhoven, Netherlands). Thermo Scientific Nicolet AVATAR 380 Fourier-transform infrared (FTIR) spectrometer (Thermo Fisher Scientific, Waltham, MA, USA) was employed on the decellularised rice straw to evaluate its structure. The crystallinity of the decellularised rice straw was measured using Thermo Scientific™ ARL™ EQUINOX 3000 diffractometer (Thermo Fisher Scientific, Waltham, MA, USA) using CuKα radiation (λ = 1.5418 Å). The crystallinity index (CI) was calculated from the obtained diffractogram using the Segal method according to the following equation:

(1) $$CI=\left[(I_{002}-I_{am}\right)/I_{002}]\times 100$$

where *I*_002_ is the maximum intensity of the crystalline peak (002 plane) at 2θ ≈ 21.9° and *I_a__m_* is the intensity of the amorphous region at 2θ ≈ 18.1°.

The water contact angle of the scaffolds was measured using a goniometer 10 s after depositing a 2 µL water droplet on 3 different spots on the scaffolds.

Atomic force microscopy (AFM) from Veeco Instruments Inc. (Plainview, NY, USA), was employed on the scaffold in its dry state (after lyophilisation) to evaluate the surface roughness of certain areas of the substrate. The hydrolytic degradation of the rice straw and its decellularised form was measured in PBS for 90 days at 37 °C, and the change in mass was recorded. The hydrolytic degradation was expressed as remaining mass. The percentage of remaining mass was calculated using Equation (2):

(2) $$\mathrm{Remaining}\;\mathrm{mass}\;(\%)\;=\;\frac{W_{t}}{W_{0}}\times 100$$

Here, $W_{t}$ and $W_{0}$ represent the weight of the specimens at time t and their initial weight, respectively. The procedure involves weighing the materials in their dry state at each time interval to avoid error due to residual water absorption. The weight of the material is constantly monitored until it reaches a constant value. The water absorption capability of the rice straw and its decellularised form was evaluated over 72 h using Equation (3).

(3) $$\mathrm{Water}\;\mathrm{absorption}\;(\%)=\frac{W_{t}-W_{0}}{W_{0}}\times 100$$

Here, $W_{t}$ and $W_{0}$ represent the weight of the wet specimens at time t and their initial weight (dry weight), respectively. In this regard, the specimens were placed in PBS at 37 °C, and at each time interval, the specimens were removed, and the unbound and free surface water was wiped with filter paper, and the weight of the specimens was recorded as the wet weight or the weight of the wet specimen at time t. The specific surface area of the decellularised scaffolds was measured by the nitrogen adsorption/desorption isotherms using a BET (Brunauer, Emmett, and Teller) analyser. Before analysis, the scaffold was degassed under vacuum at 40 °C for 48 h. Nitrogen adsorption/desorption isotherms were measured at −196 °C.

Protein adsorption on the scaffold was evaluated over 24 h at 37 °C using a protein assay kit (Thermo Scientific, Waltham, MA, USA). For this purpose, the scaffold was incubated in the culture medium (DMEM/F12), and at each time interval, the adsorbed proteins were extracted using RIPA lysate buffer. Using the kit, the amount of adsorbed protein was measured and expressed in mg/mL.

### 2.4. Biocompatibility Analysis

To evaluate the biocompatibility and potential toxicity of the scaffolds, MG63 cells were cultured on the sterilised scaffolds in 48-well plates, seeded at a density of 10,000 cells per well in 80 μL of suspension. Before cell seeding, the scaffolds were punched from the prepared decellularised samples with a diameter of 0.5 cm and were sterilised by washing three times with PBS containing 1% streptomycin/penicillin, followed by UV irradiation for 12 h. The cell-seeded plates were then incubated under standard conditions for 3, 5, and 7 days, while the culture medium (DMEM/F12) was changed every 24 h. For each time span, the corresponding plate was removed from the incubator, and once the culture medium was removed from each well, the scaffolds were washed with PBS. Then, 1 mL of cold 4% paraformaldehyde solution was added to each well for 20 min at 4 °C to fix the cells. The scaffolds were then washed three times with PBS for 15 min each and then stored at 4 °C until further analysis.

To visualise adhesion and proliferation of the MG63 cells on the scaffolds, 4′,6-diamidino-2-phenylindole (DAPI) (Fujifilm-Waco Chemicals Co., Osaka, Japan) staining was performed. For this purpose, the scaffolds were first washed twice with PBS for 5 min each. In the next step, 4 mL PBS and 4 μL DAPI stain were added to the cell-seeded scaffolds and were subsequently incubated for 12 min at room temperature. The scaffolds were then washed three times with PBS for 10 min each. The stained cells were imaged under a fluorescence microscope with a blue filter to assess the live cells present. Cell metabolic activity was assessed using the MTT (3-(4,5-Dimethylthiazol-2-yl)-2,5-Diphenyltetrazolium Bromide) assay. For each culture time, the scaffolds were incubated with 0.5 mg/mL of MTT solution at 37 °C for 4 h. The resulting formazan crystals were dissolved by adding 1 mL DMSO (Waco Co., Japan). Once the solution was collected, its optical density was measured at 570 nm using a microplate reader.

### 2.5. Evaluation of Osteogenic Differentiation

#### 2.5.1. Calcium Content Analysis

To quantify the amount of deposited calcium minerals, hydroxyapatite nanoparticles (nHA) produced by osteogenically differentiated cells seeded on the scaffolds were assessed using alizarin red staining (ARS). hMSCs were seeded at a density of 5 × 10^3^ cells per well. The hMSC cell culture process was extended for 4 weeks, and at the end of each week, ARS was performed on the scaffold. A positive control (control+) was employed using TCPS containing osteogenic differentiation factors, including ascorbic acid (250 µM), dexamethasone (12 µM), and β-glycerophosphate (10 mM). A negative control (control-) was also used, in which cells were cultured on TCPS without any osteogenic medium. The scaffold’s potential to induce osteogenesis was evaluated in the absence and presence of the osteogenic medium, which were coded as scaffold and scaffold+, respectively. For this purpose, the culture medium was gently removed, and the remaining scaffold was also gently washed with PBS three times. Then, the cell-seeded scaffolds were first fixed with 4% paraformaldehyde, followed by performing the staining process using 40 mM alizarin red stain solution Bio-idea (Ideh Zist-e Novin, Tehran, Iran) for 5 min, which was performed at room temperature. Once the process was complete, the stained scaffolds were washed three times with deionised (DI) water. The scaffolds were later lyophilised, and then the inverted phase-contrast microscope model INV-100 BEL Photonics (BEL Engineering s.r.l., Monza, Italy) was used to evaluate the surface of the scaffolds. For analysis, a 3% *v*/*v* acetic acid solution was used to dissolve possible calcium deposits on the scaffolds. Then, the solution was placed in a microplate reader, and its absorbance was measured at 540 nm. The concentration of calcium was measured by comparing the results to a standard calcium curve, which was prepared in parallel. The assay was performed in triplicate for each experimental group.

#### 2.5.2. Alkaline Phosphatase Activity

Alkaline phosphatase (ALP) activity was measured to further assess the scaffold’s osteogenic differentiation potential. ALP kit (Biorexfars Co., Shiraz, Iran) was used for this purpose. The cell-seeded scaffolds were first washed with PBS and were further incubated in 1 mL of RIPA cell lysis buffer for 20 min at 4 °C. The obtained cell lysates were then centrifuged at a rate of 12,000 rpm for 12 min, and the final supernatant was carefully transferred to a new tube. ALP activity in the lysates was determined using the ALP assay kit, and the procedure involved the kit manufacturer’s protocol. Briefly, the lysates were mixed with the p-nitrophenyl phosphate (pNPP) substrate solution and incubated at 37 °C for 30 min. The reaction was stopped by adding the stop solution, and the absorbance of the solution was recorded at a wavelength of 405 nm using a microplate reader.

#### 2.5.3. Bone Marker Gene Expression

The total RNA of the cells seeded on the scaffold with different culture times was extracted using TRIzol reagent (Yekta Tajhiz Co., Tehran, Iran) according to the manufacturer’s protocol. Briefly, the cells grown on the scaffolds were lysed directly in the culture dish by adding 1 mL of the TRIzol reagent. The cell lysate was pipetted several times to make it homogeneous. The homogenised samples were then incubated for 5 min at room temperature to allow for complete dissociation of the nucleoprotein complexes. Phase separation was performed by adding 0.2 mL of chloroform followed by centrifugation. The aqueous phase containing RNAs was then transferred to a new tube, and the RNAs were precipitated by adding isopropanol. The RNA pellet was washed once with 75% ethanol and resuspended in RNase-free water. RNA concentration and purity were determined using a Thermo Scientific NanoDrop 2000 spectrophotometer (Thermo Fisher Scientific, Waltham, MA, USA).

First-strand cDNA synthesis was performed using 1 μg of total RNA, oligo(dT) primers, and M-MuLV reverse transcriptase according to the manufacturer’s protocol. The reaction mixtures were incubated at 42 °C for 60 min, followed by enzyme inactivation at 85 °C for 5 min.

The gene-specific primers were designed using Primer 3 software based on mRNA sequences obtained from the NCBI database. The primers were synthesised by Eurofins Genomics. The primer sequences are presented in Table 2. GAPDH was used as the internal control. Relative gene expression was analysed by the 2−ΔΔCT method. The expression of the gene marker of the bone tissue was analysed by real-time polymerase chain reaction (RT-PCR) using SYBR Green chemistry on an Applied Biosystems 7500 Fast system. Each 20 μL reaction contained 12.5 μL SYBR Green mix, 1.5 μL DEPC water, 1 μL each of forward (F) and reverse (R) primers, and 9 μL cDNA template. The PCR cycling conditions were 95 °C for 10 min, followed by 40 cycles of 95 °C for 15 s and 60 °C for 1 min. The expression of osteogenic marker genes RUNX2, BMP4 and BGLAP was assessed, with GAPDH as an internal control.

#### 2.5.4. Statistical Analysis

The experiments in this study were performed in triplicate, and the statistical results were analysed using GraphPad Prism version 11.0.0 for Windows (GraphPad Software, San Diego, CA, USA, www.graphpad.com). One-way analysis of variance and Tukey’s test at a 5% significance level were used to compare the means between groups. The data are presented as mean ± standard deviation. Presence of meaningful differences between the results was represented as: ns (not significant), *p* < 0.05 (*), *p* < 0.01 (**), *p* < 0.001 (***) and *p* < 0.0001 (****).

## 3. Results

### 3.1. Physicochemical Characterization of the Decellularized Rice Straw Scaffold

**Figure 1:**
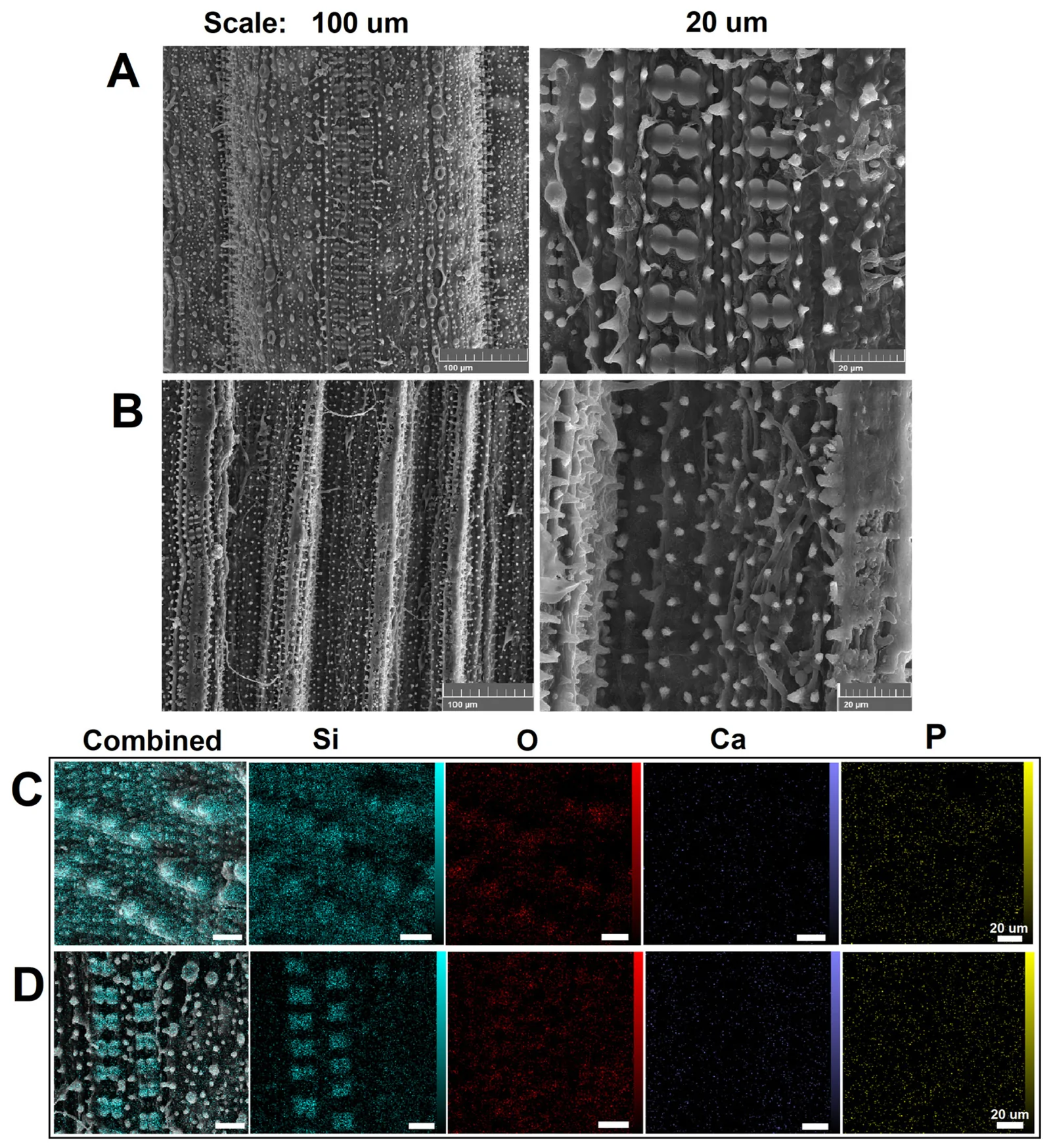
**Surface morphology and elemental composition of native and decellularised rice straw**. (**A**) FESEM micrograph of untreated rice straw. (**B**) FESEM micrograph of decellularised rice straw. Scales for (**A**,**B**): 100 µm, 20 µm, and 10 µm (as indicated). (**C**) EDX elemental mapping of untreated rice straw. (**D**) EDX elemental mapping of decellularised rice straw. Scale for (**C**,**D**): 20 µm. Both maps show the distribution of silicon (Si) (blue-green), oxygen (O) (red), calcium (Ca) (purple), and phosphorus (P) (yellow).

The surface topographies of the untreated rice straw and its decellularised state are studied by FESEM imaging, and the results are shown in Figure 1A,B, respectively. Based on Figure 1A, the plant cells of the raw rice straw are spread on the surface, and the numerous rough protrusions are used by the cells to spread. Based on the image with a scale of 100 µm, it is evident that there are major parallel fibres protruding from the surface. The fibres are more evident in Figure 1B once the plant cells are removed. The protruding spikes are also uniformly distributed throughout the surface between the major fibrous protrusions. Based on Figure 1B, the image with a scale of 20 µm, these spikes are more densely accumulated on the major fibrous protrusions. By comparing the surface topography prior to and after removing the plant cells, it is clear that the decellularisation treatment had not impacted the texture of the lignocellulosic substrate. The feature indicates that the method is not aggressive towards the lignocellulosic texture.

Figure 1C,D also represent the FESEM images of the surface alongside the elemental mapping of Si, oxygen (O), calcium (Ca) and phosphorus (P) using EDX analysis. Based on the results, it is evident that the intensity of the Si element had decreased after the treatment. It seems that the removed Si atoms have been on the plant cells, and that degradation of the cell components and washing the substrate have washed away the loose amorphous Si. Another interesting part of the analysis results is the presence of Ca, P, and O. The presence of Ca ions is crucial for the stimulation of bone regeneration. According to the literature, Ca constitutes about 0.8 to 1.7% of the rice straw [37].

The elemental composition of the untreated and decellularised rice straw was obtained from the EDX analysis. Despite the semi-quantitative nature of the test, the results can provide a rough estimate of the composition. The weight percentage (wt.%) of the elements is summarised in Table 3. Based on the results, C, O, and Si constitute the majority of elements. Si constitutes 37.45% and 29.95% of the untreated and decellularised rice straw, respectively. P comprises 5.09% and 9.45% of the untreated and decellularised rice straw, respectively, while the corresponding values for Ca are 0.61% and 1.62%. During the decellularisation process, the content of Si has experienced a considerable decrease, while the contents of P, Ca, and K showed a significant increase.

**Figure 2:**
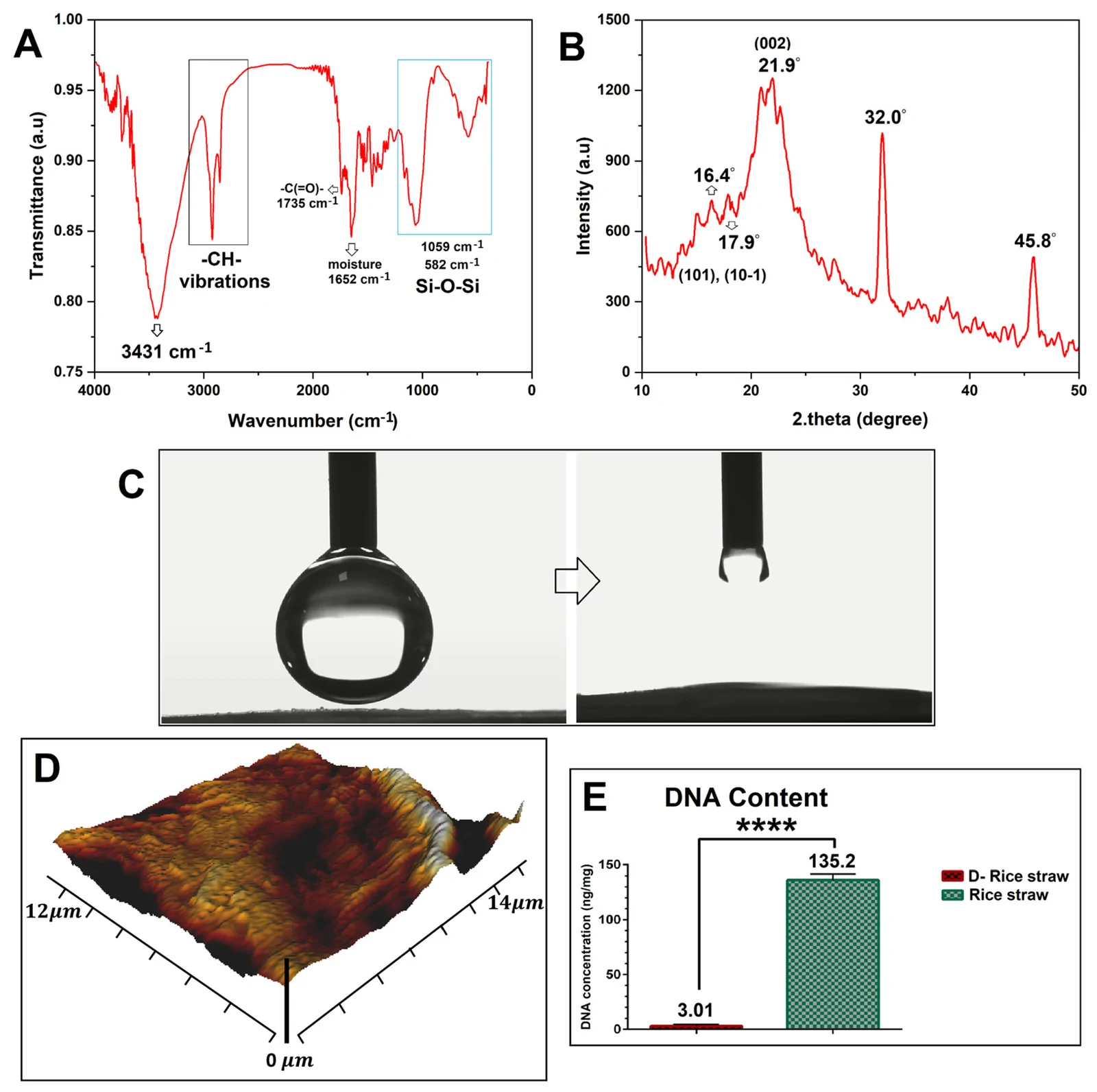
Physicochemical characterisation of decellularised rice straw. (**A**) FTIR spectrum. (**B**) XRD spectrum with crystallinity index (CI = 47%). (**C**) Water contact angle measurement before and after droplet deposition; instantaneous absorption indicates super-hydrophilicity. (**D**) AFM surface topography (scan area: 1.2 × 1.4 µm) with calculated roughness parameters (Ra = 7.2 nm, Rq = 9.6 nm). (**E**) DNA content before and after decellularisation (**** *p* < 0.0001). Data are mean ± SD (*n* = 3).

The chemical structure of the decellularised rice straw was evaluated by FTIR spectroscopy, and the result is represented in Figure 2A. The broad and relatively high-intensity peak at 3431 cm^−1^ corresponds to the stretching vibration of the hydrogen-bonded structural OH groups. The double peak present in the wavenumber range of 2900 to 3050 cm^−1^ corresponds to the -CH- groups. The relatively intense peak at 1735 cm^−1^ is also attributed to the unconjugated carbonyl group of Xylan available in the structure of hemicellulose. The absorption peak available at 1652 cm^−1^ is attributed to the absorbed moisture (H_2_O) [38]. The two peaks with a broad nature present at 1059 and 582 cm^−1^ are attributed to the stretching and bending vibrations of the Si-O-Si bond [28].

Figure 2B illustrates the XRD spectrum of the decellularised rice straw, and based on the Segal method (Equation (1)), the crystallinity index (CI) was measured. Here, the peak at 21.9° corresponds to the Miller index of (002) specific to the cellulosic component with type I nature. The term $I_{002}$ in Equation (1) corresponds to this peak. The broad and split peaks in the range of 15 to 20° correspond to the crystalline planes of (101) and (10-1), while the amorphous region was attributed to the 2θ of 18.1° [14,15]. Using the Segal method, the crystallinity index of the decellularised rice straw was calculated to be 47%.

Since Ca, P, and O are present in the treated rice straw, the sharp peaks at 32° and 45.8° could correspond to mineral salts that naturally accumulate in certain plants such as calcium phosphates (e.g., hydroxyapatite and tricalcium phosphate) or calcium carbonate that show peaks in the range of 31–33° and 45–47° [39,40]. According to the literature, rice straw stores silicon mainly as phytoliths (hydrated biogenic silica), which are amorphous in nature and could be found frequently in Si-rich plants [41,42,43].

The water contact angle test demonstrated the high hydrophilicity of the silica-enriched plant-derived scaffold. The test showed that once a water droplet contacted the scaffold surface, it immediately penetrated the scaffold without forming a contact angle. Images taken before and after the water droplet touched the scaffold surface showed no contact angle at the point of contact, only complete wetting of the scaffold. This behaviour indicates high hydrophilicity of the silica-enriched plant-derived scaffolds. Previous research results had also shown high hydrophilicity of the plant cell-derived scaffolds owing to the abundant hydroxyl groups available in each repeating unit of cellulose, hemicellulose and lignin [15,19]. As seen in Figure 1A, the spikes acted as an anchor for the plant cells to spread on the surface. To measure the roughness of the base surface, AFM analysis was performed, and the result is shown in Figure 2D with a scale of 1.2 µm × 1.4 µm. The results of roughness analysis indicated that the average roughness (R_a) was 7.196 nm and the root-mean-square roughness (R_q) was 9.621 nm. The peak-to-valley roughness was also equal to 90.95 nm. The statistical results show the surface is not very smooth, and the base surface of the substrate can also support cell adhesion [15]. The DNA content analysis also revealed that the decellularisation method removed 97.77% of the plant cell DNA content, reducing it to 3.01 ng/mg. According to the literature, the acceptable DNA content is 50 ng/mg or 95% removal of the DNA content [37]. Therefore, the decellularisation process was effective at removing the cell contents. Another feature of the substrate’s surface, which is crucial for cell attachment, is its specific surface area. The result of the BET analysis showed that the specific surface area of the substrate, measured from the BET isotherm, was 27.6255 m^2^/g.

**Figure 3:**
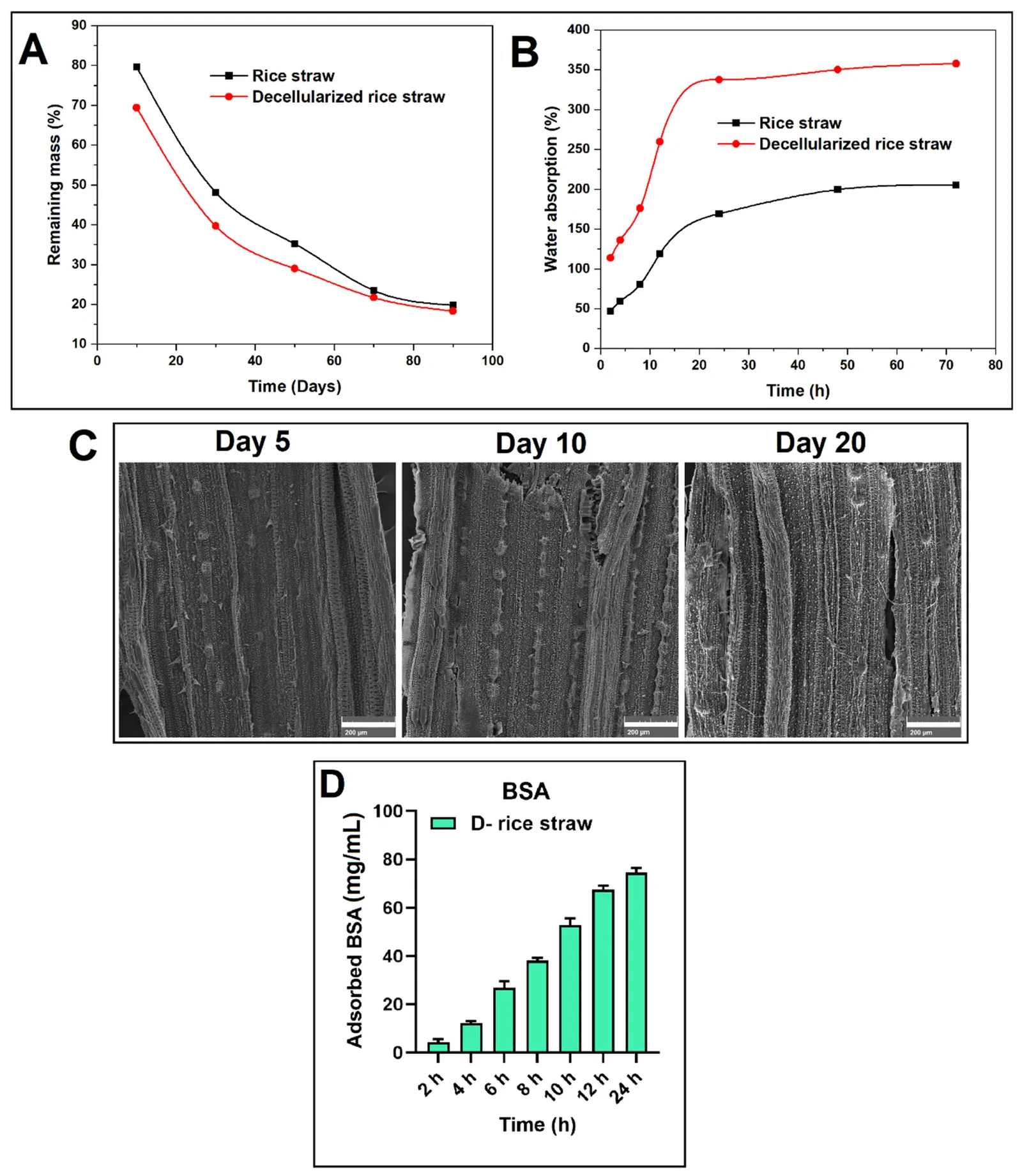
**Degradation, water uptake, and protein adsorption of scaffolds**. (**A**) Hydrolytic degradation of neat and decellularised rice straw expressed as remaining mass over 90 days in PBS (pH 7.4, 37 °C) (n = 3). (**B**) Water absorption capacity of neat and decellularised rice straw over 72 h in PBS (n = 3). (**C**) FESEM micrographs of decellularised rice straw during early degradation (days 5, 10, 20) before loss of structural integrity. Scale bar: 200 µm. (**D**) Bovine serum albumin (BSA) adsorption on decellularised rice straw over 24 h. Data are mean ± SD (n = 4).

One of the primary requirements of the scaffolds to be used for tissue regeneration is their gradual hydrolytic degradation in simulated body fluid. The hydrolytic degradation of rice straw, before and after the decellularisation process, was evaluated, and the averaged results over three samples are shown in Figure 3A as expressed by remaining mass. The decellularised rice straw showed a relatively fast degradation rate, and after 90 days, only 18.3% of its mass remained. The remaining mass can be attributed to the reported ash of rice straw, which is mainly composed of silica [38], and to the crystalline region of the cellulosic components with a denser crystalline structure. One noticeable point in the results is the marginal difference in the hydrolytic degradation extent between the two samples at each time step. The extent of hydrolytic degradation was greater for the decellularised rice straw, which could be partly due to the removal of the silica content as seen in EDX mapping. As the silica content of the rice straw in its decellularised state had decreased, parts of the difference in the degradation extent could be due to their difference in sensitivity to water. In this regard, the water absorption capability of the two samples was analysed, and the averaged results over three samples are shown in Figure 3B. Based on the results, the water uptake of the decellularised rice straw was almost two times higher than the water uptake of the untreated rice straw. The higher water adsorption of the treated rice straw could be the source of its higher hydrolytic degradation. The two samples had almost reached their water saturation after 24 h. The marginal increase in water absorption of untreated rice straw may be due to its denser, more hydrophobic nature. The fact that the water absorption of the decellularised rice straw reached 337% after 24 h indicates its high hydrophilicity. Figure 3C illustrates the FESEM micrographs of the scaffold after 5, 10 and 20 days of the degradation process, where the scaffold possesses structural integrity.

Based on the images, while the surface of the decellularised rice straw had eroded continuously, its structural (bulk) integrity had also been compromised slowly. This could be attributed to its high water-uptake capability and possible swelling, resulting in diffusion of water into the bulk of the material. The bovine serum albumin (BSA) adsorption of the scaffold was also studied, and the results over a 24 h time period were shown in Figure 3D. Based on the results, the BSA adsorption of the scaffold (D-rice straw) had continuously and steadily increased for the first 12 h, and for the next 12 h, the BSA had not increased significantly, and the final BSA adsorption was 73.1 mg/mL. The relatively high protein adsorption of the scaffold’s cellulosic nature is attributed to abundant polar hydroxyl and carbonyl groups alongside non-polar groups capable of forming hydrogen bonding and non-polar interactions with the protein [37,44].

### 3.2. In Vitro Cell Adhesion, Spreading, and Proliferation on the Scaffold

**Figure 4:**
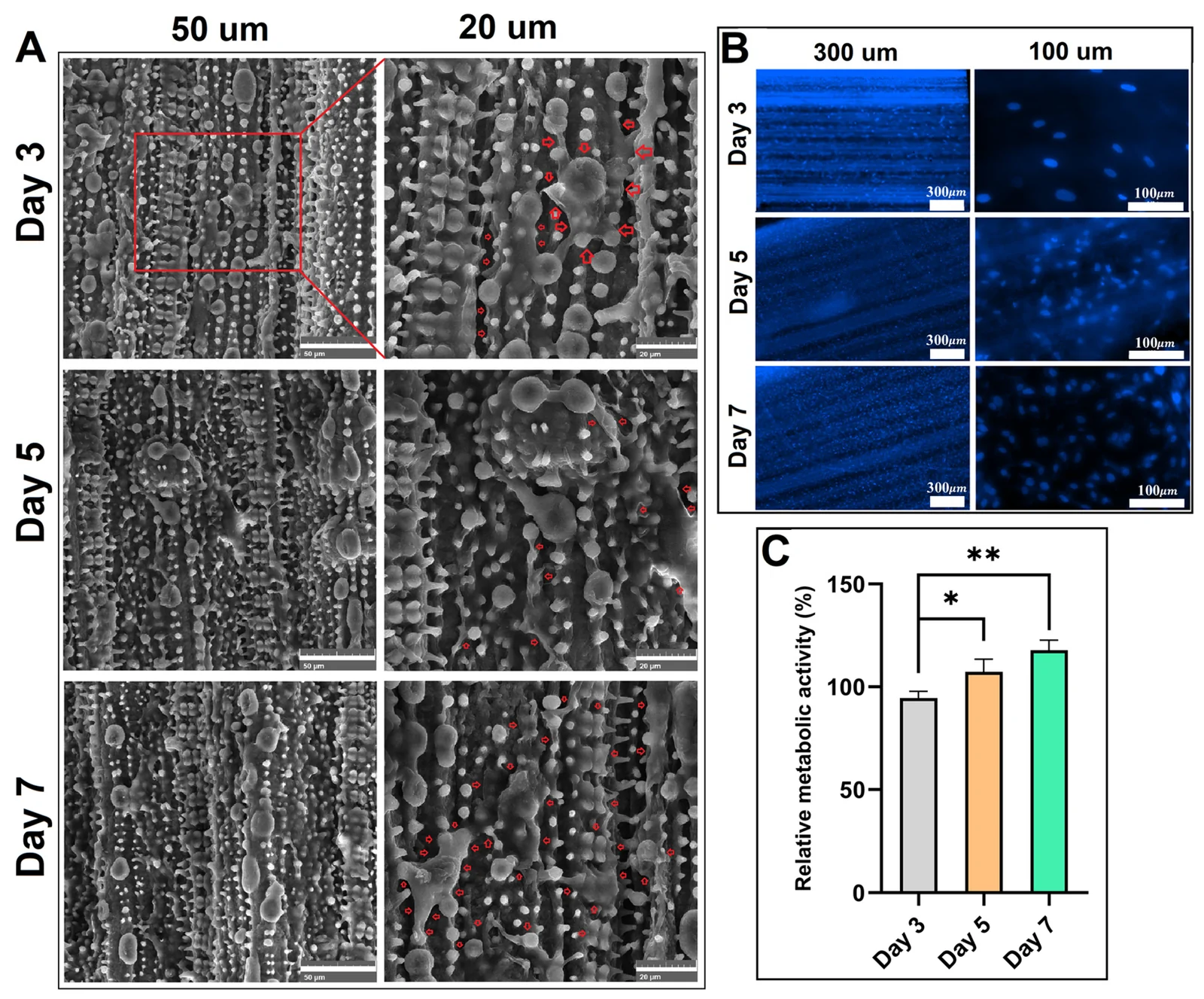
**Biocompatibility and cell proliferation on scaffolds**. (**A**) FESEM micrographs of MG63 cells cultured on decellularised rice straw for 3, 5, and 7 days. Scale bars: 50 µm and 20 µm. Red arrows indicate distinct cell regions. (**B**) DAPI staining of MG63-seeded scaffolds at days 3, 5, and 7. Scale bars: 300 µm and 100 µm. (**C**) Relative metabolic activity of MG63 cells on scaffolds compared to TCPS controls, measured by MTT assay (* *p* < 0.05, ** *p* < 0.01). Data are mean ± SD (n = 5).

Prior to evaluating the potential of the decellularised rice straw as a bioactive cellulosic scaffold, its biocompatibility was analysed using the MG63 cell line. In this regard, the cells were cultured on the scaffold for 1 week. The surface of the cell-seeded scaffold was studied after 3, 5 and 7 days of culture using FESEM imaging as a preliminary evaluation, and the results are shown in Figure 4A. Based on the micrographs of the third day of cell culture, the cells showed high adhesion to the surface, and the spikes assisted the spreading of the adhered cells, as evidenced in the 20 µm scale image. Red arrows were used to indicate certain distinct regions of the cells. After 5 days, cell growth could not be easily discerned in the micrographs; however, their distinct regions were highlighted by the red arrows. On the 7th day, the cell growth had further increased as the spikes were mostly covered and only traces of their tip could be discerned. Since the morphology of the cells could not be easily distinguished from the rough and uneven surface of the scaffold, to further evaluate the density of the live MG63 cells at the mentioned culture times, this trend was confirmed by MTT assay analysis (Figure 4B). Based on the results, as the culturing time increased to 7 days, the density of the cells on the surface increased significantly from day 3 to day 5, and the nuclei of the cells showed a bright blue colour as a sign of their metabolic activity [45]. As the culture time increased to day 7, the cells proliferated to uniformly occupy the surface and covered the whole surface of the scaffold, as evidenced in the micrograph with a scale of 300 µm. Based on the image at a scale of 100 µm for days 5 and 7 of the culturing process, clusters of MG63 cells can be identified as an indication of cell proliferation.

The metabolic activity of the MG63 cells seeded on the scaffold after 3, 5 and 7 days of cell culture was studied using the MTT assay and the results of the relative metabolic activity of the cells for each time period were reported in Figure 4C. The optical density was reported relative to that of MG63 cells cultured on the control sample (TCPS). Based on the results, the metabolic activity of the cells had increased continuously over time, and the fact that the relative metabolic activity had surpassed 100% after 5 days, with meaningful differences compared to the results of the third day, shows the unique potential of the scaffold to support the growth and proliferation of the cells. The presence of spikes and major fibrous protrusions to support the proliferation of the cells provided a higher surface area compared to the flat surface of TCPS. The spikes alongside the high surface roughness of the substrate provided an anchor for cell spreading. Hence, the scaffold was quite capable of supporting further growth and proliferation of the cells and the fact that all the available cells on the scaffold show metabolic activity proves its potential as a possible candidate for human tissue regeneration. To evaluate its potential to direct the fate of hMSCs, the growth of the stem cells over a four-week period was studied.

### 3.3. Analysing Osteogenic Differentiation

**Figure 5:**
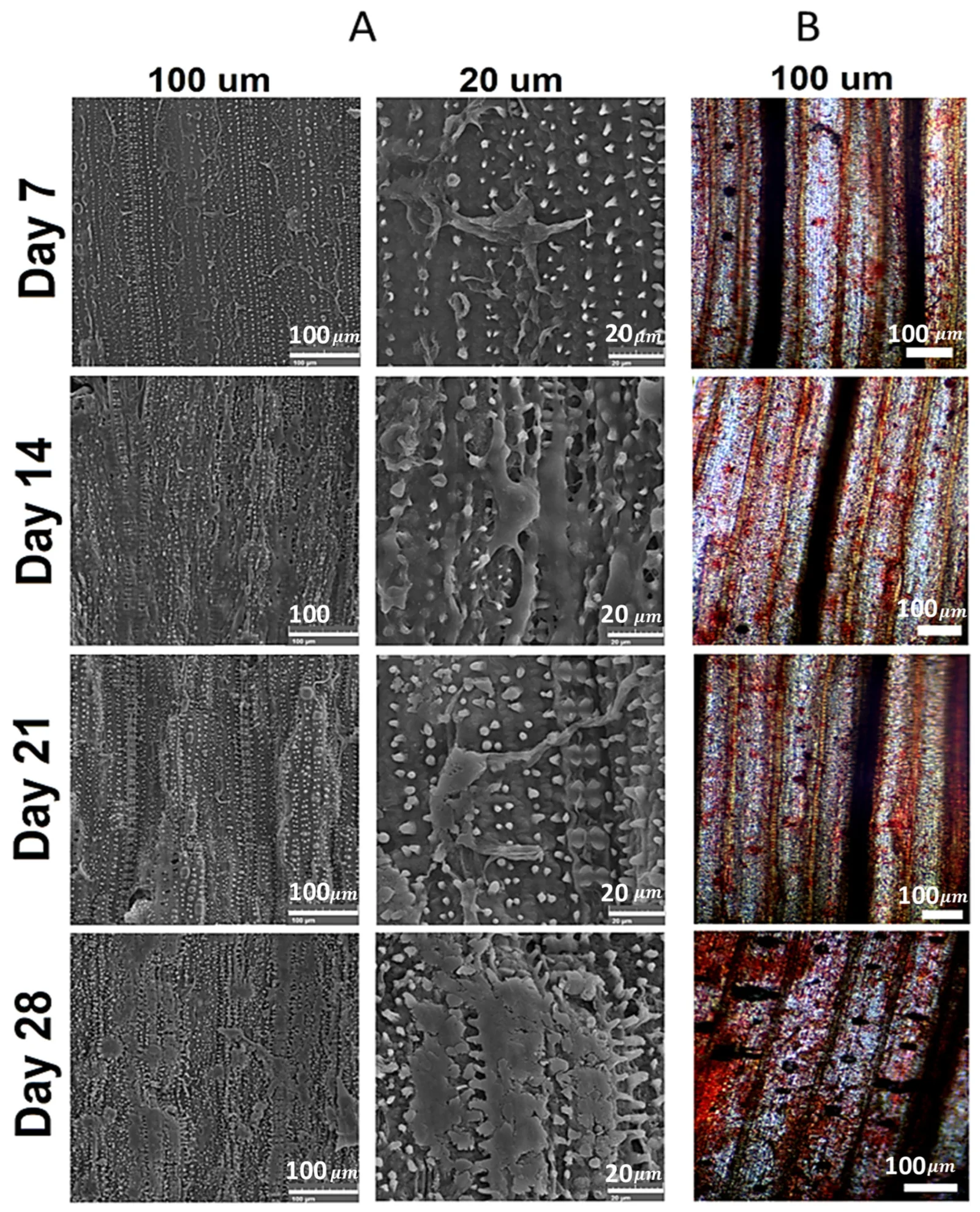
**Osteogenic differentiation of hMSCs on scaffolds without osteogenic supplements**. (**A**) FESEM micrographs of hMSC-seeded scaffolds at days 7, 14, 21, and 28. Scale bars: 100 µm and 20 µm. (**B**) Alizarin Red S staining of corresponding scaffolds showing calcium deposition. Scale bar: 100 µm. Increasing red intensity indicates progressive biomineralisation.

To evaluate the potential of the scaffold to direct the fate of hMSCs, the scaffold was seeded with hMSCs in the absence of bone-specific growth factors over a time period of 28 days. Based on the FESEM micrographs, after 14 days of cell culture, the cells have clearly spread throughout the surface while covering most of the protruded spikes. It is evident that the fibrous nature of the surface and the spikes had facilitated the proliferation of the cells. After three weeks, the cells have already undergone mineralisation, as evidenced by the micrograph with the 20 μm scale. For day 28, the calcium deposits had further accumulated on the surface, and based on the FESEM images, the deposits had grown alongside the large fibrous regions. Based on the ARS images in Figure 5, the deposition of calcium phosphate had already begun after 7 days, yet to a lesser degree compared to the third and fourth weeks, showing the high potential of the scaffold to induce biomineralisation.

The intensity of the red regions had been continuously increasing. For days 21 and 28, there are regions with a darker red colour, which could be attributed to higher accumulation of the calcium deposits as seen in the corresponding FESEM images. These results could be attributed to multiple factors, such as: (i) presence of spikes and protrusions of fibrillar structure while having high roughness on the substrate; (ii) high protein adsorption potential of the substrate; and finally, (iii) high hydrophilicity and presence of minerals such as Ca and Si-based compositions, which were shown to enhance the osteogenic differentiation of hMSCs [46,47,48,49,50,51].

We previously showed that silica nanoparticles combined with proanthocyanidin on nanofibrous scaffolds enhanced osteogenic differentiation of hMSCs, an effect they attributed to sustained silicon-ion release and topographical cues [3]. Our scaffold’s rough surface (Rq = 9.6 nm) and abundant amorphous silica produced similar outcomes without any added bioactive molecules. In another study, we demonstrated that a conductive scaffold with nanotopography promoted infection-resistant bone repair, reinforcing the idea that physical cues alone can direct stem cell fate [6]. Taken together, these reports confirm that the rice straw-derived scaffold’s intrinsic silica content and hierarchical architecture are sufficient for autonomous osteoinduction.

**Figure 6:**
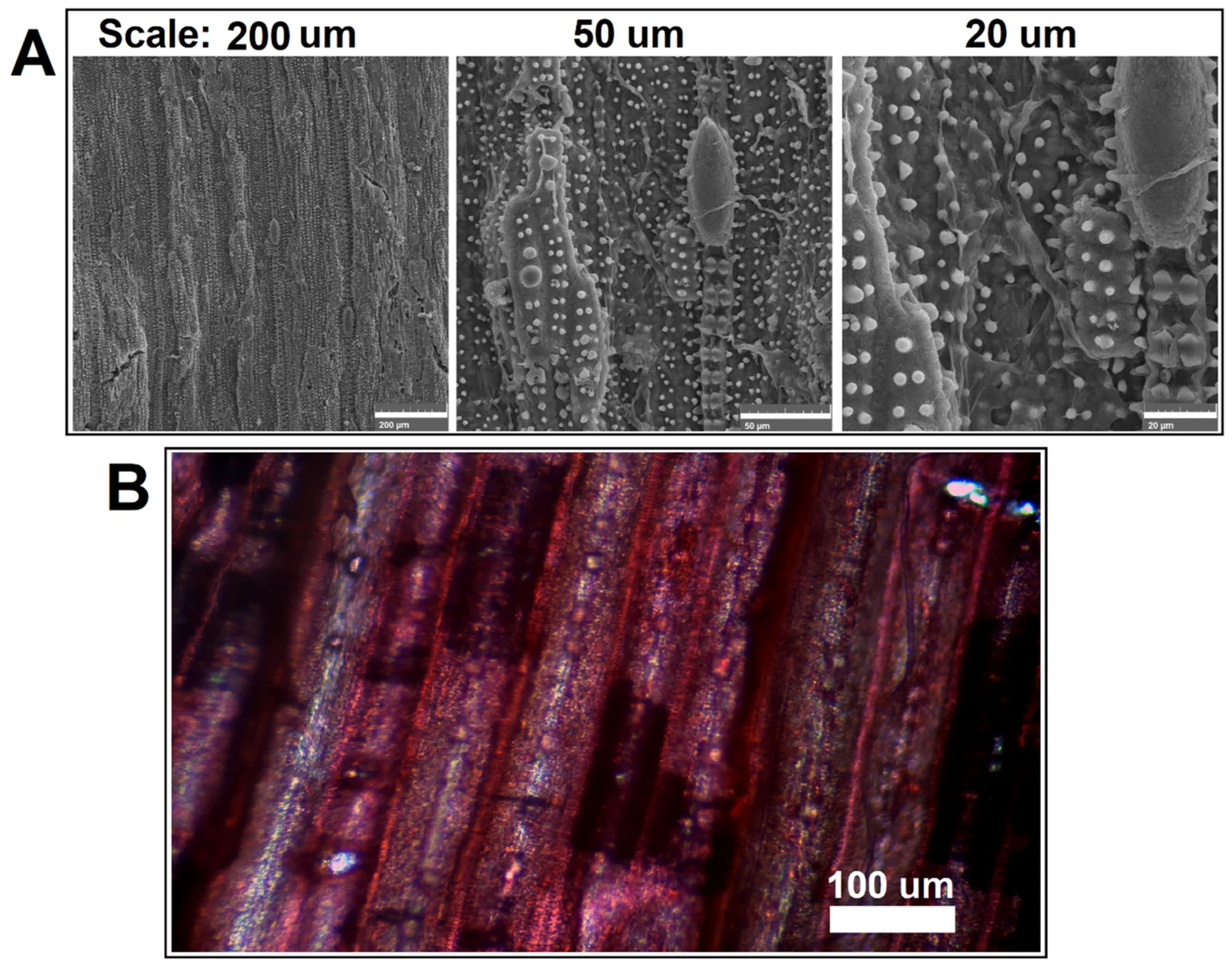
**Osteogenic differentiation of hMSCs on scaffolds with osteogenic supplements (scaffold+)**. (**A**) FESEM micrographs of hMSCs-seeded scaffolds at day 28 in the presence of differentiation factors. Scale bars: 200 µm, 50 µm, and 20 µm. Large cracks indicate brittleness from extensive mineralisation. (**B**) Alizarin Red S staining of the corresponding scaffold at day 28. Scale bar: 100 µm. Dark red regions indicate high calcium deposition.

The scaffold’s potential to induce osteogenesis in hMSCs in the presence of osteogenic medium was also evaluated. The FESEM micrographs and the ARS image of the scaffold containing osteogenic medium at day 28 of cell culture are shown in Figure 6. Based on Figure 6A, the image with a scale of 200 µm shows that the calcium deposits had completely covered the scaffold’s surface, with certain regions possessing higher accumulation of the calcium deposits. The high-intensity deposition on the surface has led to the scaffold’s brittleness, as evidenced by the large cracks observed in highly accumulated calcium deposits. The micrograph with the scales of 50 and 20 µm shows the regions with highly accumulated calcium deposits. Figure 6B shows the ARS images of the scaffold at day 28.

Comparing this image with the ARS images of the scaffold without the osteogenic medium at the same culture time shows that this scaffold had shown a greater extent of calcium deposition. The dark-red regions of the images, which are attributed to the accumulated calcium deposits, are much higher compared to the scaffold without the specific medium.

**Figure 7:**
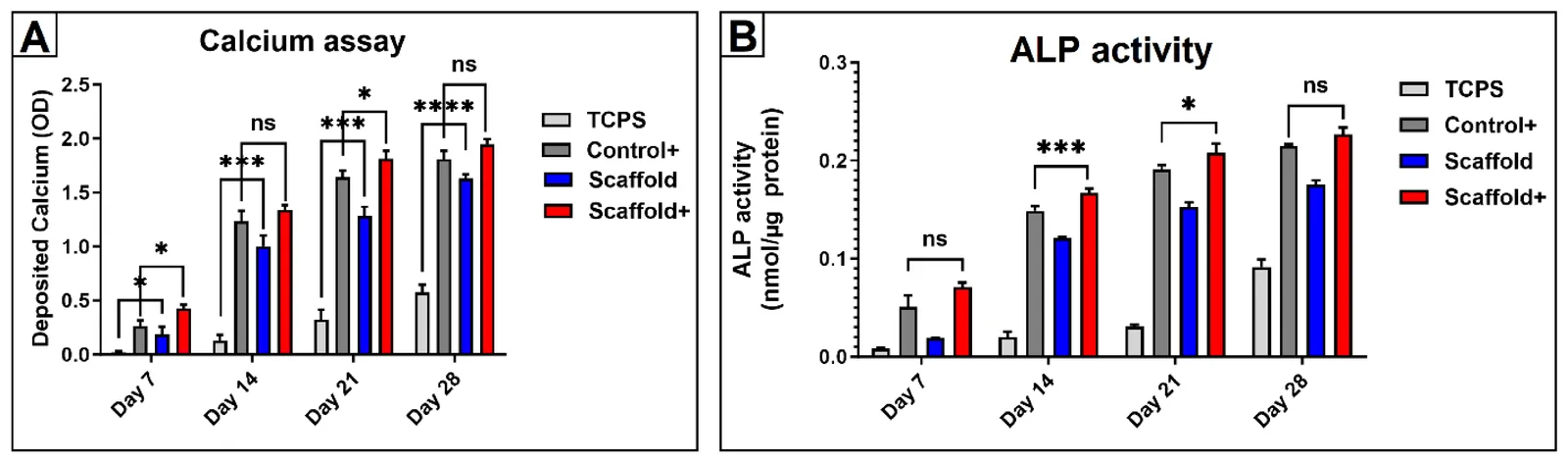
**Quantitative analysis of calcium deposition and alkaline phosphatase activity**. (**A**) Calcium content measured by Alizarin Red S quantification after 7, 14, 21, and 28 days of hMSC culture. Groups: TCPS (control−), TCPS with osteogenic medium (control+), scaffold alone (scaffold), scaffold with osteogenic medium (scaffold+). (**B**) Alkaline phosphatase (ALP) activity of hMSCs at the same time points. (ns: not significant, *: *p* < 0.05, ***: *p* < 0.001, ****: *p* < 0.0001).

In the next step, the deposition of calcium was analysed statistically, and the results are shown in Figure 7A. According to the results, there was a significant difference between the control (TCPS) and the scaffold (the scaffold without the osteogenic medium). As the culture time increased, the significance of the difference increased considerably, from *p* < 0.05 on day 7 to *p* < 0.001 on day 14 and later to *p* < 0.0001 on day 28. These results clearly show the potential of the scaffold to induce the biomineralisation of hMSCs to a large degree. The results for the scaffold supplied with the osteogenic medium (scaffold+) were compared to the results of the control+ at the end of each week. Based on the results, there was either no significant difference or, for the cases where there was a meaningful difference (day 7 and day 21), the significance of the difference was *p* < 0.05.

Alkaline phosphatase (ALP) activity of the cells during the culture period is considered a direct indicator of the biomineralisation process, as the activation level of the enzyme suppresses the biomineralisation inhibitor [51,52]. Based on Figure 7B, the ALP activity of the cells cultured on the scaffold in the absence of osteogenic medium had begun to increase intensely in the second week of the culture process. The ALP activity rose steadily over the next two weeks. In the presence of the osteogenic medium, the ALP activity of the cells cultured on the scaffold increased from the first week. For the second and third weeks of the culture process, there was a significant difference between the control+ and scaffold+ with the differences of *p* < 0.001 and *p* < 0.05, respectively. The results for the ALP activity of the cells cultured on the scaffold clearly state that the surface features of the scaffold (roughness and protrusions) are quite effective at stimulating the activation of the ALP enzyme.

**Figure 8:**
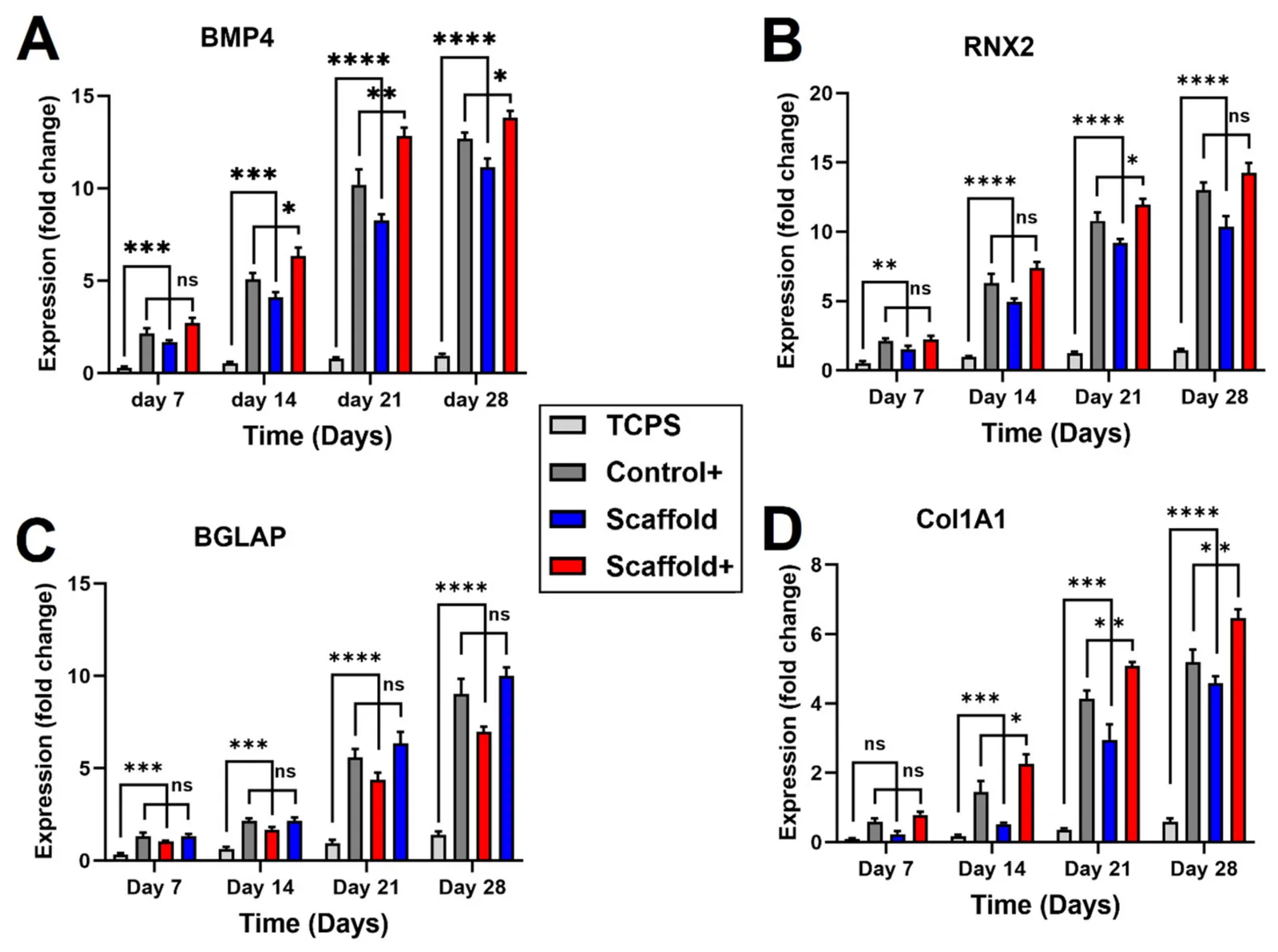
**Osteogenic gene expression profiles over four weeks of hMSC culture**. (**A**) BMP4, (**B**) RUNX2, (**C**) BGLAP (osteocalcin), and (**D**) COL1A1 (collagen type I). Relative expression was normalised to GAPDH and calculated using the 2^−^ΔΔCt method. Groups: TCPS (control−), TCPS with osteogenic medium (control+), scaffold alone (scaffold), scaffold with osteogenic medium (scaffold+) (ns: not significant, *: *p* < 0.05, **: *p* < 0.01, ***: *p* < 0.001, ****: *p* < 0.0001).

In the next step, the results of the expression levels of the bone-specific genes are studied. Bone morphogenic proteins (BMPs) are known for their capability to induce angiogenesis and osteogenesis in the bone repair process. BMP4 is one of the main proteins involved in the bone induction process. Based on Figure 8A, the changes in the expression levels of the gene over the four-week period are provided. Based on the results, the expression of BMP4 by cells cultured on the scaffold differs significantly from that on TCPS (*p* < 0.001). The significance of the difference further increased for week 3 (*p* < 0.0001). This result indicates that the scaffold was capable of activating the expression of the bone-specific gene from the early days of culture, proving its osteoinductive role. In the presence of the osteogenic medium, there has been a significant difference between the scaffold+ and the control+ from the second week of culture (*p* < 0.05). These results could be attributed to the specific surface topography of the scaffold, i.e., high roughness and presence of spikes, and the role of amorphous silicon as a soluble osteoinductive ion. Apart from these factors, the cellulosic biomaterials have been shown to act as osteoinductive materials [16,44]. RUNX2 is one of the key genes, where its activation acts as a master-switch for subsequent activation of other determining genes, which control the differentiation of the cells towards bone tissues [53,54,55,56]. RUNX2 expression (Figure 8B) differed significantly between scaffold and control samples (*p* < 0.01), with the scaffold inducing transcript levels comparable to osteogenic medium-supplemented controls by day 14. The results show the activation of the master-switch gene from the early days of the culture even in the absence of osteogenic medium.

The significance of the difference increased further to *p* < 0.0001 from the second week of culture onward. Regarding the samples supplied with osteogenic medium, there were no significant differences between the results over the four weeks except for the third week, where there was a difference of *p* < 0.05.

Figure 8C represents the results of the BGLAP gene expression, which encodes the osteocalcin protein. It is used to determine the evolution of cells towards osteogenic differentiation [57]. The results on the expression levels of the gene are relatively similar to the previous two genes, confirming the previous results on the development of osteogenic differentiation. The secretion of collagen, as a crucial part of the ECM to further improve cell adhesion, proliferation, and its evolution, was studied, and the result of the Col1A1 gene is shown in Figure 8D. According to the results, there was no significant difference between the control and the untreated scaffold, and the control+ and scaffold+ in the first week. However, from the end of the second week, the significance of the differences had increased considerably. At the end of the third week, secretion of collagen by the cells on the untreated scaffold had increased significantly. The rise in the activity of the bone-specific genes at the end of the second week and especially during the third week of culture could also be partly due to the rise in the secretion of collagen.

## 4. Limitations and Future Directions

Our in vitro data confirmed the scaffold’s osteoinductive potential, yet some aspects such as mechanical properties under load-bearing conditions require further characterisation before clinical translation. The scaffold’s high porosity and water uptake (337%) favour compliance, and similar rice-straw constructs have reported compressive moduli below 100 kPa [15,19] with 82% mass loss at 90 days. We hypothesised that silicon drives osteoinduction, but quantifying Si, Ca, or P release over time is necessary to find the mechanism of dose–response relationships [23,26]. Further examination to discover whether the scaffold triggers an immune response is needed. While plant tissues generally elicit low inflammation [13,16], detached siliceous particulates could provoke a foreign-body response. Our decellularisation protocol also uses heavy SDS and bleach, which conflicts with the material’s sustainable appeal. Moreover, time-resolved ion profiling and time-matched histological assessment in a small-animal model would resolve the most pressing unknowns before clinical trials.

## 5. Conclusions

Decellularisation of waste rice straw yields a hierarchical silica cellulose scaffold that retains the plant’s native surface architecture of abundant fibrils and spikes while preserving embedded amorphous silica, calcium, and phosphorus. This structure is highly hydrophilic, gradually degrades over 90 days, and efficiently adsorbs protein.

When seeded with MG63 cells, the scaffold supports robust adhesion, spreading, and proliferation, achieving metabolic activity levels above those on standard tissue culture plastic. More importantly, human mesenchymal stem cells cultured on the scaffold without any osteogenic supplements undergo osteogenic differentiation. Biomineralisation begins by week two and intensifies through week four, with concurrent upregulation of BMP4, RUNX2, BGLAP, and Col1A1.

The scaffold’s intrinsic osteoinductivity arises from its rough surface, protruding spikes, and soluble silicon released from the amorphous silica phase. No exogenous growth factors or synthetic additives are required. This waste-derived material offers a sustainable, low-cost, self-sufficient bone graft substitute ready for preclinical evaluation.

## Figures and Tables

**Table 1:** Summary of studies using rice straw-derived materials for bone tissue engineering applications.

Rice Straw Use	Processing Method	Final Form	Study Type	Model Used	Key Finding	Ref.
Nanocellulose extraction + TEMPO oxidation + mineralisation	Chemical extraction + mineralisation with 50 wt% biphasic calcium phosphate	Injectable hydrogel	In vitro	Osteoblast-like cells	Rice straw nanofibrillated cellulose is a nanocomposite of cellulose nanofibres and silica nanoparticles; non-toxic to osteoblast-like cells with positive alkaline phosphatase assay; BCP incorporation increased cytotoxicity	[31]
Silica extraction from rice straw ash + mesoporous wollastonite (m-WS) synthesis	Chemical synthesis	Mesoporous wollastonite (m-WS) particles	In vitro + in vivo	In vitro: mouse mesenchymal stem cells (C3H10T1/2); In vivo: rat tibial bone defect model	m-WS particles are biocompatible, stimulate MSC proliferation, promote osteoblast differentiation via Runx2 upregulation, and promote bone formation in rat tibial defects	[32]
Silica extraction from rice straw ash + mesoporous wollastonite synthesis	Chemical synthesis	Mesoporous wollastonite (m-WS) particles	In vivo	Rat tibial bone defect model	m-WS particles are bioactive and can serve as a bone substitute material for bone defect repair	[33]
Silica extraction from rice straw + wollastonite synthesis + copper doping	Sol–gel method + copper doping	Copper-doped wollastonite (Cu-Ws) particles	In vitro	Mouse mesenchymal stem cells	Cu-Ws particles are cytocompatible, enhance bone formation, and show antimicrobial effect against *E. coli* and *S. aureus*	[34]
Silica extraction from rice straw + diopside (Dp) synthesis	Chemical synthesis + composite with chitosan	CS/Dp composite scaffolds	In vitro + in vivo	In vitro: human osteoblastic cells (MG-63); In vivo: rat model	CS/Dp scaffolds are porous, biodegradable, non-cytotoxic, biocompatible, support MG-63 cell attachment and proliferation, and upregulate osteoblast differentiation marker genes	[35]

**Table 2:** The primer sequences used for the real-time polymerase chain reaction (RT-PCR) in the present study, with F and R representing the forward and reverse sequences.

Gene	Primer Sequence	Product Size
GAPDH	F: 5′-GTC TCC TCT GAC TTC AAC AGC G-3′ R: 5′-CAC CCT GTT GCT GTA GCC AA-3′	221
BGLAP	F: 5′-CAC CCT GTT GCT GTA GCC AA-3′ R: 5′-CTC TTC ACT ACC TCG CTG CC-3′	241
BMP4	F: 5′-CTG GTC TTG AGT ATC CTG AGC G-3′ R: 5′-TCA CCT CGT TCT CAG GGA TGC T-3′	201
COL1A1	F: 5′-CATCTCCCCTTCGTTTTTGAC-3′ R: 5′-CCAAATCCGATGTTTCTGCTG-3′	170
RUNX2	F: 5′-GCC TTC AAG TGG TAG CCC-3′ R: 5′-CGT TAC CCG CCA TGA GAG TA-3′	123

**Table 3:** **Quantitative** **EDX Elemental Composition of Untreated and Decellularised Rice Straw.**

Element	Wt.% (Untreated)	Wt.% (Decellularised)
C	21.92	22.11
O	32.54	33.61
Si	37.45	29.95
N	0.81	1.59
P	5.09	9.45
Ca	0.61	1.62
K	1.58	1.67

## Data Availability

The data supporting the findings of this study are available from the corresponding author upon reasonable request.

## Abbreviations

The following abbreviations are used in this manuscript:

ALP: alkaline phosphatase; ARS: alizarin red staining; BET: Brunauer, Emmett and Teller; BGLAP: bone gamma-carboxyglutamate protein (osteocalcin); BMP: bone morphogenic protein; BMP4: bone morphogenic protein 4; BSA: bovine serum albumin; Ca: calcium; cDNA: complementary deoxyribonucleic acid; Col1A1: collagen type I alpha 1; CPC: cetylpyridinium chloride; CTAB: cetyltrimethylammonium bromide; DAPI: 4′,6-diamidino-2-phenylindole; DEPC: diethyl pyrocarbonate; DI: deionized; DMEM: Dulbecco’s Modified Eagle Medium; DMSO: dimethyl sulfoxide; DNA: deoxyribonucleic acid; ECM: extracellular matrix; EDTA: ethylenediaminetetraacetic acid; EDX: energy dispersive X-ray spectroscopy; FBS: foetal bovine serum; FESEM: field-emission scanning electron microscopy; FTIR: Fourier-transform infrared; GAPDH: glyceraldehyde 3-phosphate dehydrogenase; hMSC: human mesenchymal stem cell; MTT: 3-(4,5-dimethylthiazol-2-yl)-2,5-diphenyltetrazolium bromide; O: oxygen; P: phosphorus; PBS: phosphate-buffered saline; PCL: poly(ε-caprolactone); PCR: polymerase chain reaction; PLA: polylactic acid; pNPP: p-nitrophenyl phosphate; RIPA: radioimmunoprecipitation assay; RNA: ribonucleic acid; Rq: root-mean square roughness; RSCB: Royan Stem Cell Bank; RT-PCR: real-time polymerase chain reaction; RUNX2: runt-related transcription factor 2; SDS: sodium dodecyl sulfate; SEM: scanning electron microscopy; Si: silicon; TCPS: tissue culture polystyrene; UV: ultraviolet; XRD: X-ray diffraction.
